# Increased Australian outpatient private practice psychiatric care during the COVID-19 pandemic: usage of new MBS-telehealth item and face-to-face psychiatrist office-based services in Quarter 3, 2020

**DOI:** 10.1177/1039856221992634

**Published:** 2021-02-24

**Authors:** Jeffrey CL Looi, Stephen Allison, Tarun Bastiampillai, William Pring, Rebecca Reay, Stephen R Kisely

**Affiliations:** Academic Unit of Psychiatry and Addiction Medicine, Australian National University Medical School, Canberra Hospital, Canberra, ACT, Australia; Private Psychiatry, Canberra, ACT, Australia; College of Medicine and Public Health, Flinders University, Adelaide, SA, Australia; College of Medicine and Public Health, Flinders University, Adelaide, SA, Australia; Department of Psychiatry, Monash University, Clayton, VIC, Australia; Monash University, VIC, Australia; Centre for Mental Health Education and Research at Delmont Private Hospital, Melbourne, VIC, Australia; Private Psychiatry, Melbourne, VIC, Australia; Academic Unit of Psychiatry and Addiction Medicine, Australian National University Medical School, Canberra Hospital, Canberra, ACT, Australia; Private Practice, Canberra, ACT, Australia; School of Medicine, The University of Queensland, Princess Alexandra Hospital, Woolloongabba, Brisbane, QLD, Australia; Departments of Psychiatry, Community Health and Epidemiology, Dalhousie University, Halifax, Nova Scotia, Canada

**Keywords:** COVID-19, telepsychiatry, telehealth, psychiatrist, private practice

## Abstract

**Objective:**

The Australian federal government introduced new COVID-19 psychiatrist Medicare Benefits Schedule (MBS) telehealth items to assist with providing private specialist care. We investigate private psychiatrists’ uptake of video and telephone telehealth, as well as total (telehealth and face-to-face) consultations for Quarter 3 (July–September), 2020. We compare these to the same quarter in 2019.

**Method:**

MBS-item service data were extracted for COVID-19-psychiatrist video and telephone telehealth item numbers and compared with Quarter 3 (July–September), 2019, of face-to-face consultations for the whole of Australia.

**Results:**

The number of psychiatry consultations (telehealth and face-to-face) rose during the first wave of the pandemic in Quarter 3, 2020, by 14% compared to Quarter 3, 2019, with telehealth 43% of this total. Face-to-face consultations in Quarter 3, 2020 were only 64% of the comparative number of Quarter 3, 2019 consultations. Most telehealth involved short telephone consultations of ⩽15–30 min. Video consultations comprised 42% of total telehealth provision: these were for new patient assessments and longer consultations. These figures represent increased face-to-face consultation compared to Quarter 2, 2020, with substantial maintenance of telehealth consultations.

**Conclusions:**

Private psychiatrists continued using the new COVID-19 MBS telehealth items for Quarter 3, 2020 to increase the number of patient care contacts in the context of decreased face-to-face consultations compared to 2019, but increased face-to-face consultations compared to Quarter 2, 2020.

The first confirmed case of COVID-19 in Australia was identified late January 2020. Cases rose rapidly such that on 20 March, the federal government closed the international border, followed shortly by lockdowns at state level. Although case numbers levelled and then fell, there was a second wave of infections in Victoria mid-June, necessitating a further lockdown. In response to concerns about possible mental health consequences, the federal government introduced COVID-19 Medicare Benefits Schedule (MBS) item numbers for video and telephone psychiatric consultations.^1^ This is because private psychiatric practice is mainly office-based, providing 50%–60% of specialist psychiatric care.^2^ Consequently, telehealth was rapidly adopted.^3^ Therefore, we analyse the ongoing usage of telehealth by psychiatrists during the third quarter of COVID-19 public health measures in Australia to inform contemporaneous mental health policy. We determined the amount of telehealth as well as face-to-face office-based consultations during Quarter 3, 2020, compared to the equivalent pre-COVID-19 period of Quarter 3, 2019, which was of predominantly face-to-face consultations. We also compared Quarter 3, 2020 with previously published data from Quarter 2, 2020.^3^

## Methods

MBS item service data were extracted from the Services Australia Medicare Item Reports (http://medicarestatistics.humanservices.gov.au/statistics/mbs_item.jsp) for psychiatrist practice office-based face-to-face consultations, COVID-19 video and telephone telehealth consultations for Quarter 3 (July–September) 2020, in Microsoft Excel format, and transferred to a purpose-built Excel database and analysed (totals, proportions, percentages) using Excel (Microsoft Office Home and Student 2019, Microsoft Corporation, Seattle, WA, USA). We extracted, as a baseline comparator, face-to-face consultation data from Quarter 3 (July–September), 2019 (Table 1).

**Table 1.** Overall Data Summary

Face-to-Face	F2F 2020	Video Item	VideoTele2020	Telephone Item	TeleTele2020	F2F 2019	F2F20/19	Vid+Tel2020	Vid+Tel+F2F2020	Vid/TotalTeleh2020	TotalTelheal+F2F2020/F2F2019	Telehealth2020/TotalTelehealthF2F2020	TotalTelehealth2020/F2F2019
289	38	92434	24	92474	1	94	40.43	25	63	96.00	67.02	39.68	26.60
291	6583	92435	1907	92475	1850	11,013	59.77	3757	10,340	50.76	93.89	36.33	34.11
293	1375	92436	254	92476	750	2269	60.60	1004	2379	25.30	104.85	42.20	44.25
296	21,606	92437	5531	92477	1629	32,149	67.21	7160	28,766	77.25	89.48	24.89	22.27
300	3969	91827	1466	91837	8867	6639	59.78	10,333	14,302	14.19	215.42	72.25	155.64
302	35,405	91828	9256	91838	31,562	56,196	63.00	40,818	76,223	22.68	135.64	53.55	72.64
304	104,702	91829	27,037	91839	53,592	157,698	66.39	80,629	185,331	33.53	117.52	43.51	51.13
306	89,968	91830	45,853	91840	30,962	151,558	59.36	76,815	166,783	59.69	110.05	46.06	50.68
308	5437	91831	1278	91841	1237	8531	63.73	2515	7952	50.82	93.21	31.63	29.48
342	6621	92455	830	92495	43	10,355	63.94	873	7494	95.07	72.37	11.65	8.43
344	34	92456	20	92496	8	131	25.95	28	62	71.43	47.33	45.16	21.37
346	584	92457	350	92497	80	1273	45.88	430	1014	81.40	79.65	42.41	33.78
348	6467	92458	754	92498	1105	8118	79.66	1859	8326	40.56	102.56	22.33	22.90
350	4894	92459	495	92499	424	5440	89.96	919	5813	53.86	106.86	15.81	16.89
352	9255	92460	1052	92500	2570	11,394	81.23	3622	12,877	29.04	113.02	28.13	31.79
**TOTAL**	**296,938**		**96,107**		**134,680**	**462,858**	**64.15**	**230,787**	**527,725**	**41.64**	**114.01**	**43.73**	**49.86**

*Note*. Face-to-Face: Psychiatrist Office-Based Face-to-Face MBS-Item-Number:

• New patient assessment items are telehealth items for new patients for an individual psychiatrist corresponding to face-to-face consultations 289 (assessment of new patient with autism), 291 (comprehensive assessment and 12-month treatment plan), 293 (review of 291 plan), 296 (new patient for a psychiatrist or patient not seen in last two calendar years).

• Standard office-based consultation items are time-based items for current and ongoing patients for an individual psychiatrist, corresponding to face-to-face consultations: 300 (<15 min), 302 (15–30 min), 304 (30–45 min), 306 (45–75 min) and 308 (75+ min).

• Group psychotherapy provided by a psychiatrist item equivalents: 342 (group psychotherapy 1 h plus of 2–9 unrelated patients), 344 (group psychotherapy 1 h plus of family of 3 patients) and 346 (group psychotherapy 1 h plus of family group of 2 patients).

• Items for interview of a person other than the patient, by a psychiatrist, for the care of the patient: 348 (initial diagnostic evaluation, 20–45 min), 350 (initial diagnostic evaluation, 45+ min) and 352 (20+ min, not exceeding 4 consultations).

*Totals and percentages were calculated for combined video and telephone telehealth as a proportion Quarter 3, 2019 face-to-face consultations, as well as the combined total of video–telephone telehealth and face-to-face consultations for Quarter 3, 2020. Video telehealth consultations were calculated as a percentage of total of video–telephone telehealth consultations for Quarter 3, 2020. The sum total of video–telephone telehealth and face-to-face consultations for Quarter 3, 2020 was calculated as a percentage of Quarter 3, 2019 face-to-face consultations*:

• F2F 2020: Face-to-face consultations for Quarter 3, 2020: (count)

• Video Item: Psychiatrist video telehealth MBS item number

• VideoTele2020: Psychiatrist video telehealth MBS item number services (count)

Telephone Item: Psychiatrist telephone telehealth MBS item number

• TeleTele2020: Psychiatrist telephone telehealth MBS item number services (count)

• F2F 2019: Face-to-face consultations for Quarter 3, 2019 (count)

• F2F20/19%: [(F2F 2020)] divided by (F2F 2019)] multiplied by 100: (percentage)

• Vid+Tel2020: [(VideoTele2020)] plus (TeleTele2020]: (count)

• Vid+Tel+F2F2020: [(VideoTele2020) plus (TeleTele2020) plus (F2F 2020)]: (count)

• Vid/TotalTeleh2020%: {VideoTele2020 divided by Vid+Tel2020} multiplied by 100: (percentage)

• TotalTelheal+F2F2020/F2F2019%: [(Vid+Tel+F2F2020) divided by (F2F 2019)] multiplied by 100: (percentage)

• Telehealth2020/TotalTelehealthF2F2020%: [(Vid+Tel2020) divided by (Vid+Tel+F2F2020)] multiplied by 100: (percentage)

• TotalTelehealth2020/F2F2019%: [(Vid+Tel2020) divided by (F2F 2019)] multiplied by 100: (percentage)

• Telehealth2020/TotalTelehealth+F2F2020%: [(Vid+Tel2020) divided by (Vid+Tel+F2F2020)] multiplied by 100: (percentage).

## Results

### Overall findings for Quarter 3, 2020

**Figure 1.**
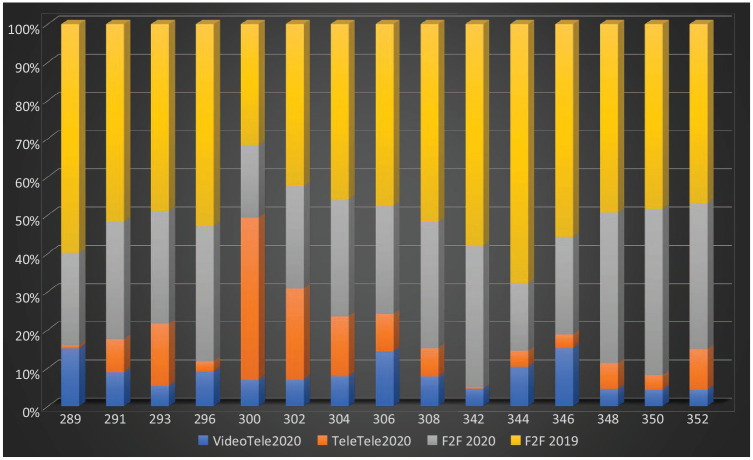
Quarter 3 individual psychiatrist MBS item usage by modality and year. *Note*. MBS-equivalent item numbers on y-axis; percentage of total consultations on x-axis; VideoTele2020: Video telehealth count; TeleTele2020: Telephone telehealth count; F2F2020: Face-to-face consultations for Quarter 3, 2020: (count); F2F 2019: Face-to-face consultations for Quarter 3, 2019 (count).

For Quarter 3, 2020, the total combined use of telehealth and face-to-face consultations increased by 14% compared to the equivalent pre-COVID-19 period in 2019 (Table 1 and Figure 1).

However, this increase masked ongoing reduction in face-to-face consultations, which were only 64% of those in the equivalent quarter of 2019. When used, face-to-face consultations were most frequently used for specific new patient for individual psychiatrist assessments (Items 289, 291, 293, 296) and longer consultations for previously seen, ongoing patients for an individual psychiatrist ⩾30 min (Items 304, 306, 308).

**Figure 2.**
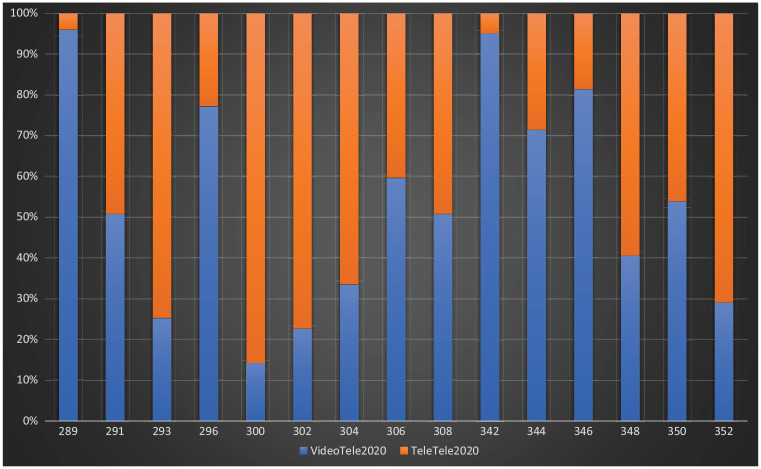
Quarter 3, 2020 video versus telephone telehealth. *Note*. MBS-equivalent item numbers on y-axis; percentage of total consultations on x-axis; VideoTele: Video telehealth consultations; TeleTele: Telephone telehealth consultations.

Video and telephone telehealth constituted 43% of the combined total of telehealth and face-to-face consultation for Quarter 3, 2020 (Figure 1). Telephone telehealth was predominantly used for shorter consultations (⩽15–30 min) with correspondingly greater video telehealth usage in longer consultations (⩾30–75 min) (Figure 2). The decrease in telehealth usage corresponds with the increase in face-to-face consultations, with an increase in video telehealth for longer consultations perhaps due to increased familiarity with video meeting platforms. Telephone telehealth remained prominent, likely due to patient and psychiatrist preferences for shorter consultations, obviating the need for transit time and travel to appointments.

## COVID-19-psychiatrist-MBS-telehealth-item usage

### New patient assessment for individual psychiatrist telehealth items

Specific MBS telehealth-equivalent items for assessment of a new patient for an individual psychiatrist were rarely used at 22%–44% of the combined total of (telehealth and face-to-face) consultations for Quarter 3, 2019 (Table 1; Figures 1–2).

For new patient assessments for an individual psychiatrist:

- Telehealth new patient assessments for autism spectrum disorders (289 equivalents) were 27% of the pre-COVID-19 Quarter 3, 2019 face-to-face consultations in the same quarter of 2019, with video telehealth used in 96% of these consultations, representing a large increase in usage of video telehealth, compared to Quarter 2, 2020.^3^
- Telehealth new patient assessment and 12-month treatment plans (291 equivalents) were 34% of 2019 face-to-face consultations, with video telehealth used in 51% of telehealth consultations representing an increased usage of video telehealth, compared to Quarter 2, 2020.^3^
- Telehealth follow-up assessment of previously new patient seen for a 12-month treatment plan (293 equivalents – patients previously seen using a 291 equivalent) were 44% of 2019 face-to-face consultations, with video telehealth used in 25% of these consultations.
- Telehealth new patient assessment items without the requirement for a 12-month treatment plan (296 equivalents) were 22% of 2019 face-to-face consultations, with video telehealth used in 77% of these consultations.

The combined total of (telehealth and face-to-face) new patient assessments for Quarter 3, 2020 was commensurate with 2019 face-to-face consultations, from the lowest of 67% for assessment for autism (289) to 89%–104% for new assessments and reviews (291, 293, 296).

### Standard office-based consultations psychiatrist telehealth items

For MBS telehealth-equivalent items to time-based office consultations for previously seen and ongoing patients of an individual psychiatrist, the majority of the overall increase in telehealth consultations comprised item 300 equivalents, that is, consultations <15 min, representing an 55% increase above the 2019 face-to-face consultations. For 300-equivalent telehealth consultations, >86% were via telephone (Table 1; Figures 1–2).

For time-based consultations of previously seen and ongoing patients of individual psychiatrists:

- Telehealth for 15–30 min (302 equivalents) were ⩾72% of the face-to-face consultations for Quarter 3, 2019. Of these consultations, 78% were by telephone.
- Telehealth for 30–45 min (304 equivalents) were 51% of the face-to-face consultations for 2019, with video used in 33% of consultations.
- Telehealth for 45–75 min (306 equivalents) were 51% of the face-to-face consultations for 2019 and use of video was 60% of all telehealth.
- Telehealth for 75 min plus (308 equivalents) were 29% of the face-to-face consultations for 2019, with video used in 51% of telehealth consultations.
- Telehealth consultations – interview of a person other than a patient to provide ongoing care of a patient – (348, 350, 352 equivalents) were used for 23%–32% compared to the face-to-face consultations for 2019, with video used in 29%–54% of telehealth consultations.

Fifteen-to-thirty-minute telehealth consultations (300–302 equivalents) represented the majority of telehealth usage. Less telehealth was used for 30–75 min (304–308 equivalents) consultations. Shorter consultations are used to provide urgent care as quantified in telephone telehealth consultations. Video telehealth may be more effective for longer consultations involving assessment, management and psychological therapy.

The combined total of (telehealth and face-to-face) standard office-based consultations for Quarter 3, 2020 equalled/exceeded Quarter 3, 2019 consultations, from the lowest of 93% for >75 min (308) to 110%–215% for items 300–306.

### Group psychotherapy psychiatrist telehealth items

Group psychotherapy telehealth consultations remained little used, likely because face-to-face consultations, were preferred for psychotherapy (Table 1; Figures 1 and 2). The combined total of (telehealth and face-to-face) group psychotherapy for Quarter 3, 2020 was between 47% and 80% of Quarter 3, 2019 face-to-face psychotherapy consultations.

## Comparison to Quarter 2, 2020 data

The 14% increase in face-to-face and telehealth consultations from Quarter 3 of 2019 to that of 2020 was identical to the increase in Quarter 2, 2020 relative to the respective 2019 quarters.^3^ However, relative proportions of face-to-face and telehealth consultations were different. The ratio of face-to-face consultations from Quarter 3 of 2020 to that of 2019 (64%) was greater than that from Quarter 2 of the same years (56%).^3^ This may be partially explained with the end of the first wave and lockdowns of the COVID-19 pandemic in Australia, with the exception of the Victorian second wave (warranting further investigation). By contrast, video and telephone telehealth use was less with comparable proportions of 43% and 51%, respectively.^3^

As in Quarter 2, 2020, face-to-face consultations were generally preferred for new patient assessment items in Quarter 3, 2020, and when telehealth was used, increasing video telehealth was used, likely to establish empathy and rapport more effectively for new patients.^3^ The combined total of (telehealth and face-to-face) new patient assessments were commensurate with Quarter 2, 2020, and Quarter 2, 2019.^3^

In comparison to Quarter 2, 2020 data,^3^ Quarter 3 results show a relative increase in face-to-face consultation, while there is maintenance of telephone telehealth for short consultations and increasing use of video telehealth for longer consultations.

Group psychotherapy telehealth was little used, similar to Quarter 2, 2020.^3^

## Discussion

Psychiatrist MBS telehealth services have formed an important part of mental healthcare during Quarter 3, 2020. This resulted in a 14% increase in the overall level of service (telehealth and face-to-face combined) compared to face-to-face-office-based consultations in Quarter 3, 2019. This increase is similar to that seen in the second quarter of 2020 compared to the same period in 2019.^3^ Higher numbers of services might have resulted from a combination of: COVID-19-related distress, shorter telehealth consultations, as well as the limited capacity for expansion of services by private psychiatrists due to existing caseload.

Telephone telehealth remains predominant for shorter consultations (⩽15–30 min) as in Quarter 2, 2020. Provision of in-depth care during new patient assessment, as well as for ongoing patients, interview of a person other than a patient, and longer consultations (⩾30–75 min) increasingly involved more video telehealth, perhaps reflecting increasing experience and confidence with telehealth technology. Overall, face-to-face consultations increased in Quarter 3, 2020 compared to Quarter 2, 2020, likely reflecting the nationally improving COVID-19 situation (with the exception of Victoria’s second wave) and a consequent return to face-to-face appointments for longer consultations and continued use of telehealth for shorter appointments.

## Implications for future private psychiatric care

These Quarter 3, 2020 results, together with those from Quarter 2, 2020,^3^ show that the private practice system adapted rapidly in Australia, mirroring the US experience.^4^ These adaptations may reduce emergency department attendance, increase the care of isolated patients and create opportunities for telehealth-enhanced shared care.^5^ Sensitivity to cultural, health and socioeconomic disparities is also needed to avoid inequities in access.^5^ Patients and psychiatrists, while still preferring face-to-face interaction, appreciate the complementary effectiveness, accessibility and convenience of telehealth, with reduced opportunity costs for consultations.^6^ The particular usefulness of shorter telephone telehealth consultations has been demonstrated during COVID-19.

## Limitations

COVID-19-psychiatrist-telehealth usage needs to be cautiously interpreted, due to jurisdictional variations in private practice. Phased introduction of COVID-19-psychiatrist-telehealth-items and restrictions to bulk billing until April 20, 2020 are likely to have limited usage by private psychiatrists, in Quarter 2, 2020, due to income reduction, and thus encouraged maintenance of face-to-face consultations, with a tailing-off effect in Quarter 3, 2020.

## Conclusions

Future research should investigate the relative proportions of newly referred and existing patients on the face-to-face and telehealth groups, as well as their demographic details such as age, gender and geographical distribution. These data should be supplemented by information on service outcomes, satisfaction with services and patient/psychiatrist consultation preferences.

Ongoing use of COVID-19-psychiatrist-MBS-telehealth-items, by patients and practitioners, beyond the first wave of the pandemic, indicates their effectiveness, complementary to face-to-face care. Furthermore, the Productivity Commission Report on Mental Health recommends making permanent the COVID-19-MBS-telehealth-consultation-items.^7^
